# The British Journals: Pathology, Practical Medicine, and Therapeutics

**Published:** 1838-10

**Authors:** 


### PATHOLOGY, PRACTICAL MEDICINE, AND THERAPEUTICS.

### *On the State of the Pupil in Typhus, and the Use of Belladonna in certain Cases of Fever.* By Robert J. Graves, m.d.

[This is an interesting and ingenious paper; and, like most of the same author's productions, is characterized by an acute spirit of observation and by originality of views. We regret that our limits permit us to make only a few extracts from it; but these will suffice to show the nature and value of the communication.]

There are two classes of narcotic remedies, which are known to possess alike the power of allaying nervous excitement, abating pain, and producing sleep; but whose effects on the state of the eye are very different, one class causing contraction, the other dilatation of the pupil. Among the former, opium holds the highest rank; among the latter, stramonium and belladonna.

It appears certain, that the state of the brain, attended with contraction of the pupil, must be very different from that which is accompanied by dilatation. In some diseases of the brain, we find the pupils of very small dimensions, in others dilated, and almost wholly insensible to the stimulus of light. It is obvious that these two opposite conditions of the pupil cannot depend on the same condition of the brain. If it be asked, on what particular conditions of that organ do they depend, the answer is, we cannot tell. Pathologists have indeed written and reasoned a great deal on this subject, but they have discovered nothing; they have found nothing on post mortem examination sufficient to explain, in a satisfactory manner, why, in one case of fever with cerebral affection, we have contraction, in another, dilatation of the pupil.

In fever with cerebral disease, one of the most alarming symptoms is marked contraction of the pupil, and were I called to a case in which every other symptom was favorable, but great contraction of the pupil present, I would say that it was a case of extreme danger. A tendency to even moderate contraction of the pupil is a very dangerous symptom in typhus, but a pupil extremely and permanently contracted, or as it has been called a pinhole pupil, is, or used to be, a fatal sign. Although this symptom is so obvious, so easily ascertained, and unhappily so common, for I have within the last few years witnessed it in a great number of fatal cases, yet, strange to say, it has not attracted the attention it deserves, among writers on typhus. No particular notice is taken of it by any author on Irish fever that I have seen. By some it may be mentioned incidentally, but by none is the state of the pupil treated of specially, and in detail.

Whenever, in attending a case of fever, you meet with this contracted state of pupil, even in a slight degree, although your patient may be restless and greatly in want of sleep, beware of opium. I have often seen it tried, and I think scarcely ever without more or less injury to the patient. When opium is administered to a patient in the advanced stage of fever, with symptoms of cerebral derangement, and tendency to contraction of the pupil, you will find that the pupil which has been moderately contracted to-day, will be greatly contracted to-morrow, and that the patient will soon sink into an irrecoverable state of coma. This contracted state of the pupil may exist in its extreme and most marked form in typhus fever, without being necessarily accompanied by headach and delirium; the patients are restless, sleepless, and in a state of remarkable nervous excitement; but they answer questions not unfrequently in a tolerably clear and rational manner, and many of them distinctly affirm that they have no pain in the head. These circumstances may deceive the unwary, but the experienced practitioner, who has witnessed many such cases, will feel that a fatal termination is threatened. Under these circumstances, opium in every shape is injurious, and even tartar emetic fails in controlling or diminishing the pernicious effects of opium. This is somewhat curious, as the combination of tartar emetic and opium seldom fails in relieving cases similar in all respects, except the symptom of contraction of the pupil.

It may appear somewhat extraordinary to think of prescribing for a single symptom, and giving belladonna in a case of fever with contraction of the pupil, merely because it produces dilatation of the pupil; but, as was before observed, it is not unreasonable to suppose that the state of the brain which accompanies dilatation of the pupil is different from that which accompanies contraction, and if belladonna has an effect in producing that cerebral state which is attended with dilatation, it is not going too far to infer, that its administration may do much towards counteracting the opposite condition; neither is it unphysiological to conclude, that if a remedy be capable of counteracting or preventing one very remarkable effect of a certain morbid state of the brain, it may also counteract other symptoms connected with the same condition. Thence it was that I was induced to try belladonna in the advanced stage of fever, accompanied by marked cerebral irritation, and a contracted state of the pupil.

I shall now proceed to make a few remarks with respect to the administration of belladonna in cases of cerebral excitement in fever, accompanied by contraction of the pupil; of stramonium I shall say nothing, having as yet no experience of its effects. I have used the belladonna by itself, or combined with musk, or with opium, or opium and tartar emetic. It is of course of the greatest consequence that the extract of belladonna employed should be extremely good. Its efficacy can be determined, and that in a very short time, (a few minutes at most,) by applying a little, mixed with a sufficient quantity of water, to render it semi-fluid to the eye of a healthy person. If it produces immediate dilatation of the pupil it can be relied on.

Stramonium is also a very powerful narcotic, and like belladonna, must be carefully watched when given internally. The dose of each should, in fever, be small, but may be repeated until the desired effect is produced. Of course belladonna cannot be expected to succeed in all cases, with contraction of the pupils, for, in several, the coexistence of other symptoms precludes the hope of recovery.

Batley's sedative, the black drop, morphia with its various salts, and in short all known preparations of opium, seem to exercise the same influence on the pupil as opium itself, and consequently are all equally objectionable in those cases where the pupil is contracted, or evinces a tendency to become so. Whether this tendency can be neutralized by combining belladonna or stramonium with them, forms a most interesting subject for clinical experiment. As far as I can judge from the result of the cases in which I have tried this combination, it promises to be a most useful addition to the remedies usually employed in fever. This combination evidently saved the life of a gentleman in Parliament-street, whom I attended along with Mr. Sibthorp and Dr. Dwyer, and whose case was apparently hopeless. In the Meath Hospital, draughts consisting of black drop, belladonna, and tartar emetic, have been eminently serviceable; the extract of belladonna which I have used, is, properly speaking, an inspissated juice, and is so called in the last edition of the Dublin Pharmacopœia.

[Two cases successfully treated on this plan are given ;and the author moreover informs us that he has "witnessed others in private practice, and in the Meath Hospital, in which belladonna seemed to produce very favorable results."]

*Dublin Journal of Med. Science. July* 1, 1838.

---

*A Statistical Enquiry on Fever, being an attempt to ascertain the prevalence, susceptibility, intensity, and prognosis, with some observations on the influence of Medical Treatment.* By ARTHUR SAUNDERS THOMSON, M.D. &c.

THIS is a very elaborate paper and most creditable to the ingenuity and industry of the author. We can only find room for the "*Concluding Results*," which are as follows:

1. That the annual ratio of deaths from fever in London, have decreased since the commencement of the eighteenth century. 2. That the susceptibility to be attacked by fever is greatest among individuals under ten years of age, and from twenty to thirty. 3. That the period of life during which the highest ratio of mortality occurs from fever is from forty to fifty. 4. That there is no very apparent difference in regard to one sex being more susceptible to fever than the other. 5. That the annual ratio of deaths by fever is nearly twice as great among the male as the female population. 6. That there is about one death for every fifteen persons attacked by fever. 7. That the intensity of fever increases with the age of the patient about thirty-four per cent. every decennial advance in life. 8. That attacks of fever are one-third more intense among males than females. 9. That fever is most prevalent from July to December inclusive. 10. That the intensity of fever is much greater during January, February, March, April, and May, than at any other part of the year. 11. That, during those months fever is most prevalent, the temperature and quantity of rain is considerably greater than during those months fever is not so prevalent. 12. That, during those months fever is most intense, the temperature and quantity of rain is comparatively low. 13. That medical treatment has a powerful effect in lessening the danger or number of deaths from fever. 14. That early medical treatment shortens the duration of fever. 15. That the mean duration of fever among individuals under forty is shorter than among those above that period of life. 16. That the general prognosis of fever is favorable, there being fourteen chances to one that the patient will recover. 17. That the prognosis of fever becomes less favorable as the patient is advanced in life, the intensity of the disease being nearly twice as great at forty-one years of age as at twenty-one. 18. That the prognosis of fever is one-third more favorable among females than males. 19. That the prognosis of fever is more favorable from June to December than from January to June. 20. That the prognosis of fever is one-half more favorable among patients who come under medical treatment before the seventh day of the disease, than those who are admitted at a later period. 21. That the prognosis of fever is unfavorable when there are cerebral or thoracic complications. 22. That the second week of fever is the most dangerous. Out of a thousand cases passing through this week eighty-two died.

*Edinburgh Med. and Surg. Journal. July* 1, 1838.

---

*On the Treatment of Rheumatic Gout.* By A. L. WIGAN, Esq., Surgeon, Brighton.

[THIS practice as recommended by a gentleman of long experience, we recommend to the notice of our readers: we ourselves have had no experience of the remedy as employed by Mr. Wigan.]

The powdered root of colchicum is the specific on which I depend. It is an old remedy, but the mode of administration is new, and entirely my own. On no one of the many occasions on which it has been used, have I seen the slightest injurious consequence; and I do not now hesitate to pronounce it the most easily managed, the most universally applicable, the safest, and the most certain specific, in the whole compass of our opulent Pharmacopœia; the mode of exhibition being an effectual regulator of its influence, adapting it accurately to the varying temperaments of different patients. Administered in the way I prescribe, and with the limitations stated, I feel quite confident that no one will be disappointed in the results to be expected from it. No doubt there may be complications of this disease, which may render other measures advisable: cases of plethora, or of inflammatory action, so decidedly marked as to leave no doubt of the propriety of taking away blood. It may be so; I have seen none such; at least none in which I have thought it necessary to use the lancet, though I see no disadvantage in erring on the safe side, and just relieving the tension of the blood-vessels by moderate depletion. Large bleedings I believe to be always injurious; they seem to me to aggravate the tendency to metastasis. The cases which require bleeding must be extremely rare, since not one has presented itself to me during the last six years. I am sorry to say, that when this disorder has gone on for some time, when it has been allowed to spend its violence on the articulations, and especially those of the vertebræ (the most frightful form of the disease), during a fortnight or three weeks, the remedy is by no means so effectual. Still it has a great influence in alleviating the pain and in shortening the duration of the malady, although the transition from intense suffering to perfect ease is less sudden and decided. I have uniformly remarked, that the more violent the attack the greater the number of articulations under its influence; the higher the fever, and the more general the disturbance, the more speedy and the more perfect is the cure. Were I to give details of some of the cases, I should not obtain belief, so much would the statement resemble a quack-doctor's advertisement. I will, however, venture to assert, that on many occasions I have seen the patient one day unable to turn in bed, with ankles and wrists so swollen and inflamed, and even the vertebræ affected to such a degree, that he has entreated me to walk gently, as the mere shaking of the bed produced an agony of suffering; I have seen, I say, a patient in this state, and thirty-six hours afterwards not only perfectly free from pain, but able to walk across the room without assistance.

If the bowels be loaded, I begin by an enema of decoct. aloes, but this does not delay the use of the colchicum. The dose, eight grains every hour, taken in the medium most acceptable to the patient—plain water, sugar and water, apple tea, ginger tea, or other analogous fluids, changing them from time to time, even with the successive doses, if necessary, according as the stomach is more or less irritable, or the palate more or less capricious. The point of saturation, as I call it, (for want of a better term,) is very uncertain, varying so much with different individuals, that although the usual quantity is eight or ten doses, I have known some take fourteen, and others unable to bear more than five. In every case it is to be repeated till active vomiting, profuse purging, or abundant perspiration take place; or at least till the stomach can bear no more. If a slight nausea comes on after three or four doses (I have never seen it so soon as the fourth), a quarter of an hour's delay may be allowed. A lump of sugar, dipped in brandy or eau de Cologne, a wine-glass of soda water, or any thing else the patient wishes for, in small quantity, may be given. Sometimes a small slice of lemon kept in the mouth will turn away the nausea, and enable him to bear a few more doses; the main object in all cases being to get into the stomach the largest quantity that it can be induced to receive. Even two doses taken at once would be rejected by a patient, who will thus gradually bear a dozen. The most usual course of things is this. At the end of the sixth or seventh dose a slight nausea comes on. By keeping quite still, turning away the thoughts by conversation, or listening to an amusing book, coaxing the palate with a slice of lemon, a clove, or some such thing, three or four more doses can be received, when the disgust becomes perhaps unconquerable. After this there is generally sound sleep, with occasional nausea on waking. The pain ceases, but the more active effects of the colchicum do not take place for some hours after the last dose. Distressing as is the state of the patient when under the full influence of the medicine, it still does not exceed an ordinary seasickness; and when this has been endured for a few hours, it is succeeded by the Elysium. The inflammation of the joints subsides, and they resume their natural size with miraculous rapidity. The acidity of the perspiration ceases, as well as the peculiar odour, which enables the experienced practitioner to recognize the disease on entering the room, before he has asked a single question. As soon as a cup of souchong tea can be retained, a sound sleep comes on, from which the patient awakes perfectly well. When circumstances will admit of it, I prefer to give a breakfast of bread and butter and tea only, very early in the morning, and two hours afterwards commence the colchicum. No more food will be required that day, but tea may be given abundantly, with bread sopped in it, if required. It will be well to indulge the returning appetite very sparingly on the day following, on which, however, we may allow a small snap of devilled meat, and rice boiled plain as for curry, which will generally be the things most acceptable to the stomach. A small quantity of good curry itself is not objectionable to those who have been accustomed to that luxury. In the subsequent treatment I have no reason to think that any precaution is necessary. The patient may resume his ordinary diet as soon as the appetite dictates. I have never seen a relapse.

The colchicum, it is obvious, should be preserved with care. The best mode, I believe, is to grind it, at the proper season, with twice or thrice its weight of fine sugar, into an impalpable powder, when, should it accidentally become damp, it is safe from injury.

It is better, if possible, that the patient should not be aware of the direct effects expected until they take place, in order that the imagination may not anticipate and interfere with the process. *Lancet, and Med. Gazette. June* 30, 1838.

---

*New Mode of Exhibiting Copaiva.* By Charles Evans, Esq.

The following is the method I have generally adopted, first premising that the article used must be good copaiva, not such an article as many druggists supply under that name:—Put any weight of copaiva into a vessel which will admit of its being conveniently stirred, and gradually add to it, constantly stirring until they are thoroughly incorporated, one-sixteenth of its weight of pure calcined magnesia; set it by for a few days, and it will then be found of sufficient consistence to admit of its being formed into pills or boluses of any shape. When about to use it, wet the hands, spatula, and slab, with water, which will prevent the mass adhering to either: form half-drachm oval boluses; then cut a slip of wafer-paper, such as confectioners use for baking biscuits on; moisten it with water, and roll the bolus in it.

Before taking one, place it in water for a minute or so, for the purpose of softening the envelope, and it will then slip down the œsophagus much easier than a small pill, and leave no trace behind it in the shape of a disagreeable taste: in fact the dose is perfectly insipid, and in this form it seems to be more grateful to the stomach, for I never have observed that loathing of the medicine and food which so frequently occurs during the ordinary exhibition of this medicine.

*Med. Gazette. July* 7, 1838.

